# Knowledge, attitudes, and practices of dietary management among patients with rheumatoid arthritis in China

**DOI:** 10.3389/fpubh.2024.1490189

**Published:** 2024-11-08

**Authors:** Xueyong Li, Ju Liu, Jie Yu, Liang Dong

**Affiliations:** ^1^Department of Rheumatology and Immunology, Jiujiang City Key Laboratory of Cell Therapy, JiuJiang NO.1 People’s Hospital, Jiujiang, China; ^2^Department of Endocrinology, Jiujiang City Key Laboratory of Cell Therapy, JiuJiang NO.1 People’s Hospital, Jiujiang, China

**Keywords:** knowledge, attitude, practice, dietary management, patients, rheumatoid arthritis

## Abstract

**Background:**

This study aims to assess the knowledge, attitudes, and practices (KAP) regarding dietary management among patients with rheumatoid arthritis (RA).

**Methods:**

A cross-sectional survey was conducted at JiuJiang NO.1 People’s Hospital from November 2023 to May 2024. Data were gathered using structured questionnaires that solicited demographic information and measured KAP scores related to dietary management in RA patients.

**Results:**

The survey yielded 504 valid questionnaires. Of the respondents, 306 (60.71%) had a Body Mass Index (BMI) within the optimal range of 18.5–23.9 kg/m^2^. The mean scores for knowledge, attitude, and practice were 10.13 ± 3.58 (possible range: 0–22), 31.38 ± 2.38 (possible range: 9–45), and 4.46 ± 2.30 (possible range: 0–12), respectively. Correlation analysis revealed significant relationships between knowledge and practice (*r* = 0.294, *p* < 0.001) as well as between attitude and practice (*r* = 0.178, *p* < 0.001). Multivariate logistic regression showed that knowledge score (OR = 1.165, 95% CI: [1.078, 1.259], *p* < 0.001) was independently associated with proactive practice. The structural equation model (SEM) results showed that knowledge had direct effects on attitude (*β* = 0.291, *p* < 0.001) and practice (*β* = 0.188, *p* < 0.001). Meanwhile, attitude had a direct impact on practice (*β* = 0.081, *p* = 0.045).

**Conclusion:**

This study indicates that patients with RA generally demonstrate inadequate knowledge but hold positive attitudes toward dietary management, despite relatively inactive practices in implementing such dietary changes. Healthcare providers should prioritize educational interventions that not only enhance patient understanding but also actively support the implementation of dietary management strategies in clinical settings.

## Introduction

Rheumatoid arthritis (RA) is a chronic systemic autoimmune disease characterized by unknown etiology and significant joint damage. It globally affects approximately 460 individuals per 100,000 population (1), with a prevalence of 0.32–0.42% within the Chinese population (2). If RA is not adequately treated, it can lead to persistent synovitis and the erosion of articular cartilage and surrounding bone, resulting in reduced mobility, decreased quality of life, and complications including cardiovascular and other extra-articular issues (3, 4). In addition to these complications, RA is often associated with metabolic comorbidities such as diabetes. Studies have shown that patients with RA are at an increased risk of developing type 2 diabetes due to chronic inflammation and glucocorticoid use, which can impair insulin sensitivity and glycemic control. Managing these comorbidities requires a holistic approach, where diet plays a pivotal role not only in mitigating RA symptoms but also in controlling blood glucose levels in diabetic RA patients (5, 6).

Dietary management is essential in RA treatment, as specific foods can help reduce inflammation and improve patient outcomes (7). For instance, diets rich in anti-inflammatory components, such as omega-3 fatty acids, have been shown to alleviate RA symptoms, while unhealthy choices may worsen them (8). Understanding how RA patients approach dietary management is vital for effective care.

Management of RA involves a comprehensive approach that includes both pharmacologic and non-pharmacologic (9, 10). Pharmacologic treatments primarily consist of disease-modifying anti-rheumatic drugs (DMARDs), nonsteroidal anti-inflammatory drugs (NSAIDs), and glucocorticoids, which help manage the inflammation and pain associated with the disease (4). Complementarily, non-pharmacologic treatments such as patient education, physiotherapy, and nutritional therapy play critical roles in the overall management of RA (11).

The Knowledge-Attitude-Practice (KAP) model is a crucial framework in the healthcare domain, extensively used to assess the knowledge, attitudes, and practices of target populations (12). This model is founded on the premise that enhanced knowledge positively influences attitudes, which in turn, shape individual practices, a process integral to health literacy (13). The use of the KAP questionnaire alongside this model helps evaluate the demand and level of acceptance of health-related content (14).

Diet, as a key modifiable factor in chronic disease management, significantly impacts patient outcomes, with robust evidence supporting the benefits of dietary improvements (15). For patients with rheumatoid arthritis (RA), dietary choices are critical as they can influence symptoms significantly; certain foods, especially those rich in n-3 polyunsaturated fatty acids (PUFAs) from fish, have been shown to alleviate symptoms, while unhealthy options may exacerbate them. Additionally, evidence suggests that dietary management can play a crucial role in preventing and managing diabetes in RA patients, with anti-inflammatory and low-glycemic diets helping control both inflammation and blood sugar levels (16, 17).

Despite the established importance of diet in RA management, there is a notable gap in research focusing on the KAP regarding dietary management among these patients. While there have been studies examining KAP in patients with chronic conditions like diabetes, the specific area of dietary KAP in RA patients remains underexplored. By exploring how RA patients perceive and implement dietary recommendations, researchers can pinpoint knowledge gaps and barriers to effective management. This understanding is essential for creating targeted educational programs and interventions that enhance dietary habits, thereby improving the quality of life and health outcomes. Therefore, this study aims to assess the KAP regarding dietary management among patients with RA, addressing an underserved area of research with potential significant clinical implications.

## Methods

### Study design and participants

This cross-sectional study was conducted from November 2023 to May 2024 at JiuJiang NO.1 People’s Hospital. The study participants were patients with rheumatoid arthritis (RA). The study was approved by the Ethics Committee of JiuJiang NO.1 People’s Hospital (Ethic No. JJSDYRMYY-YXLL-2023-217), and written informed consent was obtained from all participants.

Inclusion criteria: Patients diagnosed with rheumatoid arthritis (meeting the 1987 ACR or 2010 ACR/EULAR criteria), who can communicate effectively in the local language, possess sufficient reading and writing skills, and are willing to participate.

Exclusion criteria: Patients with cognitive impairments, severe organic diseases or complications, or concurrent malignant tumors.

Questionnaire distribution: The survey was conducted via the Wenjuanxing platform and disseminated using WeChat and WeChat groups.

### Questionnaire introduction

The questionnaire was developed in accordance with established guidelines and relevant literature (8, 18, 19), enriched by insights from multiple experts in the specified field. Prior to full implementation, it underwent a preliminary test involving a small cohort of 47 participants. The results from this pre-test demonstrated a robust overall reliability coefficient of 0.801. Specifically, the reliability coefficients for the knowledge, attitudes, and practices sections were 0.898, 0.680, and 0.759, respectively, indicating satisfactory internal consistency across both the overall questionnaire and its discrete sections.

The final version of the questionnaire, written in Chinese, comprised four distinct dimensions for collecting information, totaling 48 items. These dimensions included: Basic Information with 16 items, Knowledge with 11 items, Attitudes with 9 items, and Practices with 12 items.

For statistical analysis, the scoring for each item varied according to the dimension it belonged to. In the Knowledge Dimension (items K1 to K11), scoring was as follows: “Very familiar” earned 2 points, “Somewhat familiar” earned 1 point, and “Not familiar” received 0 points, with the total possible score ranging from 0 to 22 points. For the Attitude Dimension, scoring differed based on the nature of the statement: positive items (A1–A3, A7–A9) scored from 5 points for “Strongly agree” to 1 point for “Strongly disagree,” while negative items (A4–A6) scored inversely, with “Strongly agree” earning 1 point and “Strongly disagree” 5 points, leading to a total score range of 9–45 points. The Practice Dimension simply assigned 1 point for “Yes” responses and 0 points for “No,” with a potential score range from 0 to 12 points. A scoring threshold exceeding 60% for each dimension was set to determine adequate knowledge, positive attitudes, and proactive practices (20).

### Sample size calculation

The sample size was calculated based on the minimum sample size formula for cross-sectional studies:


$$n={\left(\frac{Z_{1}-a/2}{dx}\right)}^{2}\times p\times \left(1-p\right)$$

Where

- *α* = 0.05.
-
$Z_{1}-a/2$ = 1.96.
- *δ* = 0.05.
- *p* = 0.5

$$n={\left(\frac{1.96}{0.05}\right)}^{2}\times 0.5\times \left(1-0.5\right)\approx 384$$

Using these values, the calculated minimum sample size was 384. This sample size is adequate to ensure a robust representation of the population and provide sufficient statistical power for the analysis. Considering an 80% effective response rate, at least 480 questionnaires were planned to be collected to account for potential non-responses or incomplete data.

### Statistical analysis

Data analysis was conducted using SPSS 27.0 (IBM, Armonk, NY, USA) and AMOS 26.0 (IBM, Armonk, NY, USA). Continuous data are presented as means and standard deviations (SD), while categorical data are expressed as *n* (%). Continuous variables underwent a normality test, with the t-test for normally distributed data and the Wilcoxon Mann–Whitney test for non-normally distributed data when comparing two groups. For three or more groups with normally distributed continuous variables and uniform variance, ANOVA was used for comparisons, while the Kruskal–Wallis test was employed for non-normally distributed data. Univariate and multivariate logistic regression were performed to explore the risk factors associated with K, A, and P, with 60% of the total score was used as the cut-off value. Structural equation modeling (SEM) was utilized to explore the relationships between knowledge (K), attitude (A), and practice (P). Model fit was evaluated using root mean square error of approximation (RMSEA), incremental fit index (IFI), Tucker–Lewis index (TLI), and comparative fit index (CFI). A two-sided *p*-value less than 0.05 was considered statistically significant.

## Results

Initially, this study collected a total of 507 questionnaires. Of these, one was excluded due to the respondent being underage (0.20%), and two were excluded due to incorrect age data (0.39%), resulting in 504 valid questionnaires. The validity rate was 99.41%. Among the respondents, 407 (80.75%) were female, with a mean age of 59.72 ± 12.36 years. A total of 306 participants (60.71%) reported a BMI within the range of 18.5–23.9 kg/m^2^. Regarding socioeconomic status, 287 respondents (56.94%) reported an average monthly income of 2,000–5,000 yuan, and 210 (41.67%) had attained an elementary school level education or lower. Furthermore, 174 participants (34.52%) had been diagnosed with rheumatoid arthritis for less than 5 years. Morning stiffness of less than 15 min was reported by 314 respondents (62.30%), while 296 (58.73%) were in the remission phase of the disease, 140 (27.78%) presented with joint deformity, and 494 (98.02%) were currently receiving pharmacological treatment as their primary form of management. The mean scores for knowledge, attitude, and practice were 10.13 ± 3.58, 31.38 ± 2.38, and 4.46 ± 2.30, respectively. Variations in knowledge scores were significantly associated with average monthly income (*p* < 0.001), educational attainment (*p* = 0.028), duration of rheumatoid arthritis (*p* = 0.037), joint function classification (*p* = 0.002), disease stage (*p* = 0.001), and pain assessment (*p* = 0.002). Attitude scores varied significantly across variables such as average monthly income (*p* = 0.012), type of medical insurance (*p* = 0.020), morning stiffness (*p* = 0.003), joint function classification (*p* < 0.001), joint deformity (*p* < 0.001), pain assessment (*p* < 0.001), and history of surgical treatment (*p* = 0.003). Practice scores showed significant variation based on average monthly income (*p* < 0.001), past year residence (*p* < 0.001), education level (*p* < 0.001), and medical insurance type (*p* = 0.011) (Table 1).

**Table 1:** Baseline characteristics.

	*N* (%)	Knowledge		Attitude		Practice	
		Mean ± SD	*p*	Mean ± SD	*p*	Mean ± SD	*p*
Total	504	10.13 ± 3.58		31.38 ± 2.38		4.46 ± 2.30	
Age	59.72 ± 12.36						
Gender			0.736		0.712		0.932
Male	97 (19.25)	10.24 ± 3.10		31.30 ± 2.45		4.47 ± 2.20	
Female	407 (80.75)	10.10 ± 3.68		31.40 ± 2.36		4.45 ± 2.33	
BMI			0.072		0.736		0.737
<18.5	62 (12.30)	10.71 ± 3.75		31.31 ± 2.53		4.42 ± 2.27	
18.5–23.9	306 (60.71)	9.84 ± 3.38		31.33 ± 2.21		4.41 ± 2.28	
≥24.0	136 (26.98)	10.51 ± 3.88		31.51 ± 2.66		4.59 ± 2.37	
Average monthly income (in RMB)			<0.001		0.012		<0.001
<2,000	140 (27.78)	9.61 ± 2.98		31.14 ± 2.27		3.79 ± 2.26	
2,000–5,000	287 (56.94)	9.99 ± 3.24		31.64 ± 2.36		4.48 ± 1.96	
>5,000	77 (15.28)	11.57 ± 5.12		30.84 ± 2.51		5.58 ± 3.04	
Marital status			0.122		0.407		0.089
Unmarried	7 (1.39)	7.57 ± 5.47		32.00 ± 2.52		5.43 ± 3.05	
Married	453 (89.88)	10.15 ± 3.55		31.40 ± 2.39		4.49 ± 2.29	
Divorced	12 (2.38)	11.58 ± 4.08		30.33 ± 2.53		4.83 ± 3.01	
Widowed	32 (6.35)	9.84 ± 3.18		31.28 ± 1.99		3.56 ± 1.85	
Residence in the past year			0.566		0.096		<0.001
Rural	166 (32.94)	9.89 ± 3.15		31.16 ± 2.43		3.97 ± 2.28	
Urban	303 (60.12)	10.24 ± 3.73		31.56 ± 2.33		4.78 ± 2.25	
Suburban	35 (6.94)	10.31 ± 4.14		30.89 ± 2.43		3.97 ± 2.42	
Education level			0.028		0.058		<0.001
Elementary school and below	210 (41.67)	9.80 ± 3.03		31.60 ± 2.32		4.07 ± 2.06	
Middle school	145 (28.77)	9.85 ± 2.98		31.28 ± 2.39		4.26 ± 2.27	
High school/Technical school	84 (16.67)	10.88 ± 3.93		31.52 ± 2.58		5.00 ± 2.16	
Associate degree/Bachelor’s degree or above	65 (12.90)	10.85 ± 5.36		30.71 ± 2.14		5.46 ± 2.88	
Medical insurance type			0.780		0.020		0.011
Out-of-pocket/State-funded/Commercial insurance	29 (5.75)	10.28 ± 4.70		30.10 ± 2.78		4.69 ± 3.47	
Urban employee basic medical insurance	197 (39.09)	10.21 ± 3.57		31.49 ± 2.34		4.80 ± 2.24	
Urban resident basic medical insurance	106 (21.03)	10.30 ± 3.90		31.26 ± 2.41		4.47 ± 2.32	
New rural cooperative medical system	172 (34.13)	9.90 ± 3.15		31.53 ± 2.27		4.01 ± 2.07	
Presence of Comorbidities (e.g., hypertension, hyperlipidemia, diabetes, heart disease such as atrial fibrillation, malignancies, metabolic syndrome, etc.)			0.747		0.203		0.486
Yes	178 (35.32)	10.20 ± 3.37		31.20 ± 2.35		4.36 ± 2.09	
No	326 (64.68)	10.09 ± 3.69		31.48 ± 2.39		4.51 ± 2.41	
Duration of rheumatoid arthritis			0.037		0.111		0.959
<5 years	174 (34.52)	9.60 ± 3.52		31.67 ± 2.38		4.46 ± 2.22	
6–10 years	128 (25.40)	10.37 ± 3.52		31.42 ± 2.30		4.37 ± 2.34	
11–20 years	129 (25.60)	10.74 ± 3.86		31.21 ± 2.34		4.52 ± 2.48	
>20 years	73 (14.48)	9.89 ± 3.13		30.92 ± 2.50		4.49 ± 2.15	
Morning stiffness			0.114		0.003		0.710
<15 min	314 (62.30)	10.37 ± 3.68		31.39 ± 2.31		4.52 ± 2.35	
15–60 min	176 (34.92)	9.78 ± 3.40		31.52 ± 2.46		4.34 ± 2.12	
>60 min	14 (2.78)	9.07 ± 2.84		29.29 ± 2.02		4.57 ± 3.41	
Joint function classification			0.002		<0.001		0.576
Level I	385 (76.39)	10.14 ± 3.26		31.72 ± 2.31		4.51 ± 2.21	
Level II	91 (18.06)	10.41 ± 4.24		30.40 ± 2.26		4.34 ± 2.63	
Level III	23 (4.56)	7.91 ± 4.50		30.26 ± 2.18		3.91 ± 2.00	
Level IV	5 (0.99)	14.00 ± 4.64		27.80 ± 0.84		5.00 ± 4.53	
Disease stage			0.001		0.284		0.697
Remission phase	296 (58.73)	10.55 ± 3.79		31.28 ± 2.34		4.49 ± 2.39	
Active phase	208 (41.27)	9.52 ± 3.16		31.51 ± 2.43		4.41 ± 2.18	
Joint deformity			0.209		<0.001		0.833
Yes	140 (27.78)	10.45 ± 4.35		30.74 ± 2.47		4.42 ± 2.49	
No	364 (72.22)	10.00 ± 3.23		31.63 ± 2.29		4.47 ± 2.23	
Pain assessment			0.002		<0.001		0.124
0	19 (3.77)	11.26 ± 6.31		29.68 ± 2.52		5.05 ± 2.88	
1–3	421 (83.53)	10.31 ± 3.35		31.56 ± 2.31		4.52 ± 2.27	
4–6	59 (11.71)	8.59 ± 3.62		30.81 ± 2.37		3.85 ± 2.28	
7–10	5 (0.99)	8.60 ± 3.21		29.20 ± 3.11		4.20 ± 2.39	
History of surgical treatment			0.145		0.003		0.194
Yes	47 (9.33)	10.85 ± 4.46		30.40 ± 2.37		4.87 ± 2.89	
No	457 (90.67)	10.05 ± 3.47		31.48 ± 2.36		4.41 ± 2.24	
Current main treatment method			0.548		0.270		0.528
Drug therapy	494 (98.02)	10.11 ± 3.49		31.36 ± 2.36		4.47 ± 2.31	
Other	10 (1.98)	10.80 ± 6.71		32.20 ± 3.22		4.00 ± 2.21	

The distribution of knowledge dimensions shown that the three questions with the highest number of participants choosing the “Not clear” option were “Although vegetables are rich in antioxidants, some such as potatoes, tomatoes, and eggplants contain glycoalkaloids that can increase intestinal permeability, potentially adversely affecting the relief of rheumatoid arthritis symptoms.” (K11) with 53.97%, “Assessment of weight and body mass is based on body mass index (BMI).” (K3) with 40.28%, and “Are you familiar with the anti-inflammatory diet?” (K10) with 18.25% (Table 2).

**Table 2:** Knowledge dimension.

	Very knowledgeable *N* (%)	Heard about it *N* (%)	Not clear *N* (%)
1. Rheumatoid arthritis is a common chronic inflammatory autoimmune disease. Diet, as an external factor, plays a central role in disease risk and progression.	45 (8.93)	439 (87.1)	20 (3.97)
2. Increased body mass index (BMI) is associated with an increased risk of rheumatoid arthritis. Weight management helps prevent or control rheumatoid arthritis, while also reducing joint pressure and delaying joint damage.	36 (7.14)	433 (85.91)	35 (6.94)
3. Assessment of weight and body mass is based on body mass index (BMI).	33 (6.55)	268 (53.17)	203 (40.28)
4. Rheumatoid arthritis patients should supplement 3–6 g of omega-3 polyunsaturated fatty acids daily, consume fish such as mackerel, salmon, and sardines twice a week, and may also consider adding fish oil and some vegetable oils.	29 (5.75)	396 (78.57)	79 (15.67)
5. Rheumatoid arthritis patients should consume more bread, fruits, vegetables, and fish, and less red meat. They should use products based on vegetable oils instead of butter and cheese.	39 (7.74)	425 (84.33)	40 (7.94)
6. Rheumatoid arthritis patients should regularly assess vitamin D3 levels and supplement when necessary.	54 (10.71)	391 (77.58)	59 (11.71)
7. Rheumatoid arthritis patients should follow a low-salt diet with a daily salt intake of less than 5 g.	52 (10.32)	414 (82.14)	38 (7.54)
8. Exercise is an important part of nutritional support for obese patients.	146 (28.97)	326 (64.68)	32 (6.35)
9. Are you familiar with the Mediterranean diet? (The Mediterranean diet is considered an anti-inflammatory diet, mainly consisting of extra virgin olive oil, whole grains, fish, fruits, and vegetables, among other components.)	30 (5.95)	385 (76.39)	89 (17.66)
10. Are you familiar with the anti-inflammatory diet? (The anti-inflammatory diet is based on the principles of the Mediterranean diet and emphasizes foods rich in antioxidants, polyphenols, carotenoids, and omega-3 fatty acids (long chain), all of which have anti-inflammatory properties that can modify the inflammatory processes and pathways of rheumatoid arthritis.)	31 (6.15)	381 (75.6)	92 (18.25)
11. Although vegetables are rich in antioxidants, some such as potatoes, tomatoes, and eggplants contain glycoalkaloids that can increase intestinal permeability, potentially adversely affecting the relief of rheumatoid arthritis symptoms.	24 (4.76)	208 (41.27)	272 (53.97)

Responses to the attitudinal dimension showed that 37.10% disagreed that they would prefer to be treated with diet management (A3), 28.37% agreed that diet management would affect their quality of life (A4), and 21.83% agreed that diet management was too burdensome and changed habits, which was difficult for them (A6) (Table 3).

**Table 3:** Attitude dimension.

	Strongly agree *N* (%)	Agree *N* (%)	Neutral *N* (%)	Disagree *N* (%)	Strongly disagree *N* (%)
1. You consider diet management to be very important for improving rheumatoid arthritis symptoms.	36 (7.14)	426 (84.52)	39 (7.74)	3 (0.60)	0
2. You are willing to obtain various information related to diet management for rheumatoid arthritis disease through various sources such as the internet, television, books, etc.	33 (6.55)	416 (82.54)	45 (8.93)	7 (1.39)	3 (0.60)
3. You believe that diet management has fewer side effects compared to drug or surgical treatments, and therefore, you are more willing to accept diet management as a treatment.	30 (5.95)	141 (27.98)	143 (28.37)	187 (37.10)	3 (0.60)
4. You think that diet management for rheumatoid arthritis will affect your quality of life.	21 (4.17)	143 (28.37)	97 (19.25)	230 (45.63)	13 (2.58)
5. You think that diet management can help manage rheumatoid arthritis, even though it may not cure it.	10 (1.98)	68 (13.49)	74 (14.68)	258 (51.19)	94 (18.65)
6. Diet management for rheumatoid arthritis may seem cumbersome and a change from your previous lifestyle, making it difficult for you.	9 (1.79)	110 (21.83)	114 (22.62)	192 (38.10)	79 (15.67)
7. You feel confident in consistently choosing the right foods in your daily life and avoiding foods that worsen the disease.	18 (3.57)	415 (82.34)	53 (10.52)	17 (3.37)	1 (0.20)
8. Diet management for rheumatoid arthritis requires patient explanations and guidance from doctors and nurses to develop good habits and increase confidence, which is crucial for preventing heart disease.	31 (6.15)	435 (86.31)	33 (6.55)	5 (0.99)	0
9. You believe that actively cooperating with the doctor’s treatment plan and timely communication with medical staff are important for improving the disease.	42 (8.33)	443 (87.90)	17 (3.37)	1 (0.20)	1 (0.20)

The distribution of practice dimensions shown that the three questions with the highest number of participants choosing the “No” option were “You primarily use olive oil for cooking.” (P1) with 92.06%, “You use 4 tablespoons or more of olive oil daily.” (P2) with 91.47%, and “You consume less than 1 tablespoon of butter or margarine or cream daily.” (P5) with 89.68% (Table 4).

**Table 4:** Practice dimension.

	Yes *N* (%)	No *N* (%)
1. You primarily use olive oil for cooking.	40 (7.94)	464 (92.06)
2. You use 4 tablespoons or more of olive oil daily.	43 (8.53)	461 (91.47)
3. You eat 2 servings or more of vegetables daily.	454 (90.08)	50 (9.92)
4. You eat 3 servings or more of fruits daily.	251 (49.80)	253 (50.20)
5. You consume less than 1 tablespoon of butter or margarine or cream daily.	52 (10.32)	452 (89.68)
6. You drink less than 1 serving of sugary or sweetened beverages daily.	187 (37.10)	317 (62.90)
7. You eat 3 servings or more of legumes weekly.	283 (56.15)	221 (43.85)
8. You eat 3 servings or more of fish or seafood weekly.	148 (29.37)	356 (70.63)
9. You eat 1 serving or more of nuts weekly.	170 (33.73)	334 (66.27)
10. You consume poultry (chicken or turkey) more frequently than red meat (beef, veal, pork, hamburgers, or sausages).	95 (18.85)	409 (81.15)
11. You eat red meat and processed meat only once a week or 1–2 times a week.	363 (72.02)	141 (27.98)
12. You consume candy or pastries in total less than 3 servings per week.	160 (31.75)	344 (68.25)

Correlation analysis revealed significant relationships between knowledge and practice (*r* = 0.294, *p* < 0.001) as well as between attitude and practice (*r* = 0.178, *p* < 0.001) (Table 5).

**Table 5:** Correlation analysis.

	Knowledge	Attitude	Practice
Knowledge	1		
Attitude	−0.013 (*p* = 0.764)	1	
Practice	0.294 (*p* < 0.001)	0.178 (*p* < 0.001)	1

A cutoff value representing the top 60% of scores for each dimension was used to classify the participants, with 465 (92.26%) falling below this threshold for knowledge, 26 (5.16%) for attitude, and 460 (91.27%) for practice (Supplementary Table S1). Multivariate logistic regression showed that with average monthly income (OR = 3.875, 95% CI: [1.19, 12.613], *p* = 0.025), had rheumatoid arthritis for 11–20 years (OR = 2.751, 95% CI: [1.015, 7.456], *p* = 0.047), level II joint function (OR = 3.147, 95% CI: [1.176, 8.423], *p* = 0.022), level IV joint function (OR = 34.712, 95% CI: [3.121, 386.072], *p* = 0.004), pain assessment score 1–3 (OR = 0.157, 95% CI: [0.042, 0.581], *p* = 0.006), and pain assessment score 4–10 (OR = 0.101, 95% CI: [0.013, 0.768], *p* = 0.027) were independently associated with knowledge (Table 6). Concurrently, knowledge score (OR = 1.155, 95% CI: [1.017, 1.311], *p* = 0.026), with urban employee basic medical insurance (OR = 5.402, 95% CI: [1.373, 21.251], *p* = 0.016), with new rural cooperative medical system (OR = 8.678, 95% CI: [1.934, 38.934], *p* = 0.005), level II joint function (OR = 0.163, 95% CI: [0.044, 0.610], *p* = 0.007), level IV joint function (OR = 0.017, 95% CI: [0.001, 0.217], *p* = 0.002), pain assessment score 1–3 (OR = 5.085, 95% CI: [1.056, 24.487], *p* = 0.043) were independently associated with attitude (Table 7). Moreover, knowledge score (OR = 1.165, 95% CI: [1.078, 1.259], *p* < 0.001) was independently associated with proactive practice (Table 8).

**Table 6:** Univariate AND multivariate logistic regression analysis for knowledge.

	Univariate analysis		Multivariate analysis	
	OR (95% CI)	*p*	OR (95% CI)	*p*
Age	0.960 (0.935, 0.985)	0.002	0.978 (0.948, 1.009)	0.169
Gender				
Male	Ref.			
Female	1.097 (0.469, 2.566)	0.831		
BMI				
<18.5	Ref.			
18.5–23.9	0.491 (0.196, 1.232)	0.130		
≥24.0	0.902 (0.345, 2.358)	0.833		
Average monthly income (in RMB)				
<2,000	Ref.		Ref.	
2,000–5,000	0.846 (0.346, 2.067)	0.714	1.131 (0.408, 3.137)	0.813
>5,000	4.675 (1.912, 11.430)	0.001	3.875 (1.19, 12.613)	0.025
Marital status				
Unmarried	Ref.			
Married	0.747 (0.278, 2.003)	0.562		
Residence in the past year				
Rural	Ref.			
Urban	1.212 (0.578, 2.541)	0.610		
Suburban	1.818 (0.543, 6.083)	0.332		
Education level				
Elementary school and below	Ref.		Ref.	
Middle school	0.712 (0.261, 1.943)	0.508	0.349 (0.106, 1.155)	0.085
High school/Technical school	1.980 (0.802, 4.890)	0.139	1.526 (0.533, 4.371)	0.431
Associate degree/Bachelor’s degree or above	3.736 (1.588, 8.790)	0.003	1.319 (0.389, 4.472)	0.656
Medical insurance type				
Out-of-pocket/State-funded/Commercial insurance	Ref.			
Urban employee basic medical insurance	0.478 (0.146, 1.567)	0.223		
Urban resident basic medical insurance	0.651 (0.188, 2.250)	0.498		
New rural cooperative medical system	0.427 (0.126, 1.446)	0.171		
Presence of Comorbidities (e.g., hypertension, hyperlipidemia, diabetes, heart disease such as atrial fibrillation, malignancies, metabolic syndrome, etc.)				
Yes	0.801 (0.395, 1.622)	0.537		
No	Ref.			
Duration of rheumatoid arthritis				
<5 years	Ref.		Ref.	
6–10 years	1.240 (0.489, 3.147)	0.650	1.434 (0.48, 4.285)	0.519
11–20 years	2.322 (1.017, 5.302)	0.046	2.751 (1.015, 7.456)	0.047
>20 years	0.951 (0.288, 3.135)	0.934	0.797 (0.179, 3.548)	0.766
Morning stiffness				
<15 min	Ref.			
≥15 min	0.471 (0.218, 1.014)	0.054		
Joint function classification				
Level I	Ref.		Ref.	
Level II	1.705 (0.791, 3.675)	0.174	3.147 (1.176, 8.423)	0.022
Level III	0.628 (0.081, 4.842)	0.655	1.779 (0.186, 17.014)	0.617
Level IV	9.205 (1.472, 57.553)	0.018	34.712 (3.121, 386.072)	0.004
Disease stage				
Remission phase	Ref.			
Active phase	0.465 (0.221, 0.976)	0.043	0.475 (0.188, 1.2)	0.115
Joint deformity				
Yes	1.913 (0.979, 3.740)	0.058		
No	Ref.			
Pain assessment				
0	Ref.			
1–3	0.166 (0.059, 0.469)	<0.001	0.157 (0.042, 0.581)	0.006
4–10	0.107 (0.024, 0.482)	0.003	0.101 (0.013, 0.768)	0.027
History of surgical treatment				
Yes	2.324 (0.964, 5.602)	0.060		
No	Ref.			
Current main treatment method				
Drug therapy	Ref.			
Other	5.452 (1.352, 21.987)	0.017	3.076 (0.542, 17.465)	0.205

**Table 7:** Univariate AND multivariate logistic regression analysis for attitude.

	Univariate analysis		Multivariate analysis	
	OR (95% CI)	*p*	OR (95% CI)	*p*
Knowledge score	1.153 (1.024, 1.297)	0.018	1.155 (1.017, 1.311)	0.026
Age	0.989 (0.958, 1.022)	0.521		
Gender				
Male	Ref.			
Female	1.943 (0.819, 4.069)	0.132		
BMI				
<18.5	Ref.			
18.5–23.9	1.977 (0.678, 5.762)	0.212		
≥24.0	1.404 (0.440, 4.478)	0.567		
Average monthly income (in RMB)				
<2,000	Ref.			
2,000–5,000	1.448 (0.604, 3.474)	0.407		
>5,000	1.254 (0.373, 4.213)	0.715		
Marital status				
Unmarried	Ref.			
Married	1.168 (0.338, 4.036)	0.806		
Residence in the past year				
Rural	Ref.			
Urban	1.721 (0.742, 3.990)	0.206		
Suburban	0.757 (0.200, 2.868)	0.682		
Education level				
Elementary school and below	Ref.			
Middle school	0.856 (0.330, 2.225)	0.750		
High school/Technical school	0.790 (0.262, 2.384)	0.676		
Associate degree/Bachelor’s degree or above	1.033 (0.276, 3.873)	0.961		
Medical insurance type				
Out-of-pocket/State-funded/Commercial insurance	Ref.		Ref.	
Urban employee basic medical insurance	4.922 (1.489, 16.265)	0.009	5.402 (1.373, 21.251)	0.016
Urban resident basic medical insurance	2.946 (0.86, 10.093)	0.085	2.360 (0.583, 9.564)	0.229
New rural cooperative medical system	5.764 (1.632, 20.354)	0.007	8.678 (1.934, 38.934)	0.005
Presence of Comorbidities (e.g., hypertension, hyperlipidemia, diabetes, heart disease such as atrial fibrillation, malignancies, metabolic syndrome, etc.)				
Yes	0.732 (0.329, 1.630)	0.445		
No	Ref.			
Duration of rheumatoid arthritis				
<5 years	Ref.		Ref.	
6–10 years	1.031 (0.320, 3.326)	0.959	0.544 (0.143, 2.065)	0.371
11–20 years	1.040 (0.322, 3.352)	0.948	0.604 (0.152, 2.400)	0.474
>20 years	0.298 (0.107, 0.834)	0.021	0.226 (0.051, 1.009)	0.051
Morning stiffness				
<15 min	Ref.			
≥15 min	0.816 (0.367, 1.816)	0.619		
Joint function classification				
Level I	Ref.		Ref.	
Level II	0.261 (0.109, 0.624)	0.003	0.163 (0.044, 0.610)	0.007
Level III	0.338 (0.071, 1.608)	0.173	0.369 (0.043, 3.150)	0.362
Level IV	0.048 (0.007, 0.316)	0.002	0.017 (0.001, 0.217)	0.002
Disease stage				
Remission phase	Ref.			
Active phase	0.811 (0.367, 1.791)	0.604		
Joint deformity				
Yes	0.427 (0.192, 0.947)	0.036	2.352 (0.580, 9.536)	0.231
No	Ref.		Ref.	
Pain assessment				
0	Ref.		Ref.	
1–3	4.746 (1.255, 17.954)	0.022	5.085 (1.056, 24.487)	0.043
4–10	1.527 (0.354, 6.586)	0.570	3.372 (0.553, 20.562)	0.188
History of surgical treatment				
Yes	0.544 (0.179, 1.650)	0.282		
No	Ref.			
Current main treatment method				
Drug therapy	Ref.			
Other	–	0.999		

**Table 8:** Univariate AND multivariate logistic regression analysis for practice.

	Univariate analysis		Multivariate analysis	
	OR (95% CI)	*p*	OR (95% CI)	*p*
Knowledge score	1.218 (1.133, 1.310)	<0.001	1.165 (1.078, 1.259)	<0.001
Attitude score	0.997 (0.875, 1.136)	0.964		
Age	0.969 (0.945, 0.993)	0.001	0.986 (0.958, 1.015)	0.346
Gender				
Male	Ref.			
Female	1.286 (0.555, 2.978)	0.558		
BMI				
<18.5	Ref.			
18.5–23.9	2.138 (0.631, 7.238)	0.222		
≥24.0	1.731 (0.465, 6.437)	0.413		
Average monthly income (in RMB)				
<2,000	Ref.		Ref.	
2,000–5,000	1.039 (0.437, 2.469)	0.931	0.643 (0.237, 1.740)	0.384
>5,000	5.405 (2.238, 13.056)	<0.001	1.686 (0.543, 5.233)	0.366
Marital status				
Unmarried	Ref.			
Married	0.687 (0.275, 1.713)	0.420		
Residence in the past year				
Rural	Ref.			
Urban	1.714 (0.816, 3.601)	0.155		
Suburban	2.013 (0.593, 6.831)	0.262		
Education level				
Elementary school and below	Ref.		Ref.	
Middle school	1.654 (0.655, 4.179)	0.287	1.079 (0.363, 3.208)	0.891
High school/Technical school	2.680 (1.025, 7.008)	0.044	1.371 (0.408, 4.608)	0.610
Associate degree/Bachelor’s degree or above	7.293 (3.042, 17.482)	<0.001	2.127 (0.562, 8.048)	0.266
Medical insurance type				
Out-of-pocket/State-funded/Commercial insurance	Ref.		Ref.	
Urban employee basic medical insurance	0.482 (0.177, 1.312)	0.153	0.603 (0.189, 1.929)	0.394
Urban resident basic medical insurance	0.356 (0.115, 1.099)	0.073	0.427 (0.116, 1.575)	0.201
New rural cooperative medical system	0.163 (0.050, 0.526)	0.002	0.280 (0.069, 1.140)	0.076
Presence of Comorbidities (e.g., hypertension, hyperlipidemia, diabetes, heart disease such as atrial fibrillation, malignancies, metabolic syndrome, etc.)				
Yes	0.664 (0.333, 1.324)	0.245		
No	Ref.			
Duration of rheumatoid arthritis				
<5 years	Ref.			
6–10 years	0.802 (0.339, 1.894)	0.614		
11–20 years	1.188 (0.544, 2.592)	0.665		
>20 years	1.124 (0.438, 2.884)	0.807		
Morning stiffness				
<15 min	Ref.			
≥15 min	0.939 (0.494, 1.786)	0.848		
Joint function classification				
Level I	Ref.			
Level II	1.362 (0.643, 2.883)	0.420		
Level III	0.501 (0.065, 3.842)	0.506		
Level IV	2.758 (0.299, 25.417)	0.371		
Disease stage				
Remission phase	Ref.			
Active phase	0.887 (0.470, 1.673)	0.710		
Joint deformity				
Yes	1.386 (0.719, 2.672)	0.329		
No	Ref.			
Pain assessment				
0	Ref.		Ref.	
1–3	0.361 (0.114, 1.145)	0.084	0.648 (0.175, 2.398)	0.516
4–10	0.184 (0.037, 0.913)	0.038	0.544 (0.094, 3.155)	0.498
History of surgical treatment				
Yes	1.986 (0.832, 4.744)	0.122		
No	Ref.			
Current main treatment method				
Drug therapy	Ref.			
Other	1.165 (0.144, 9.418)	0.886		

**Figure 1:**
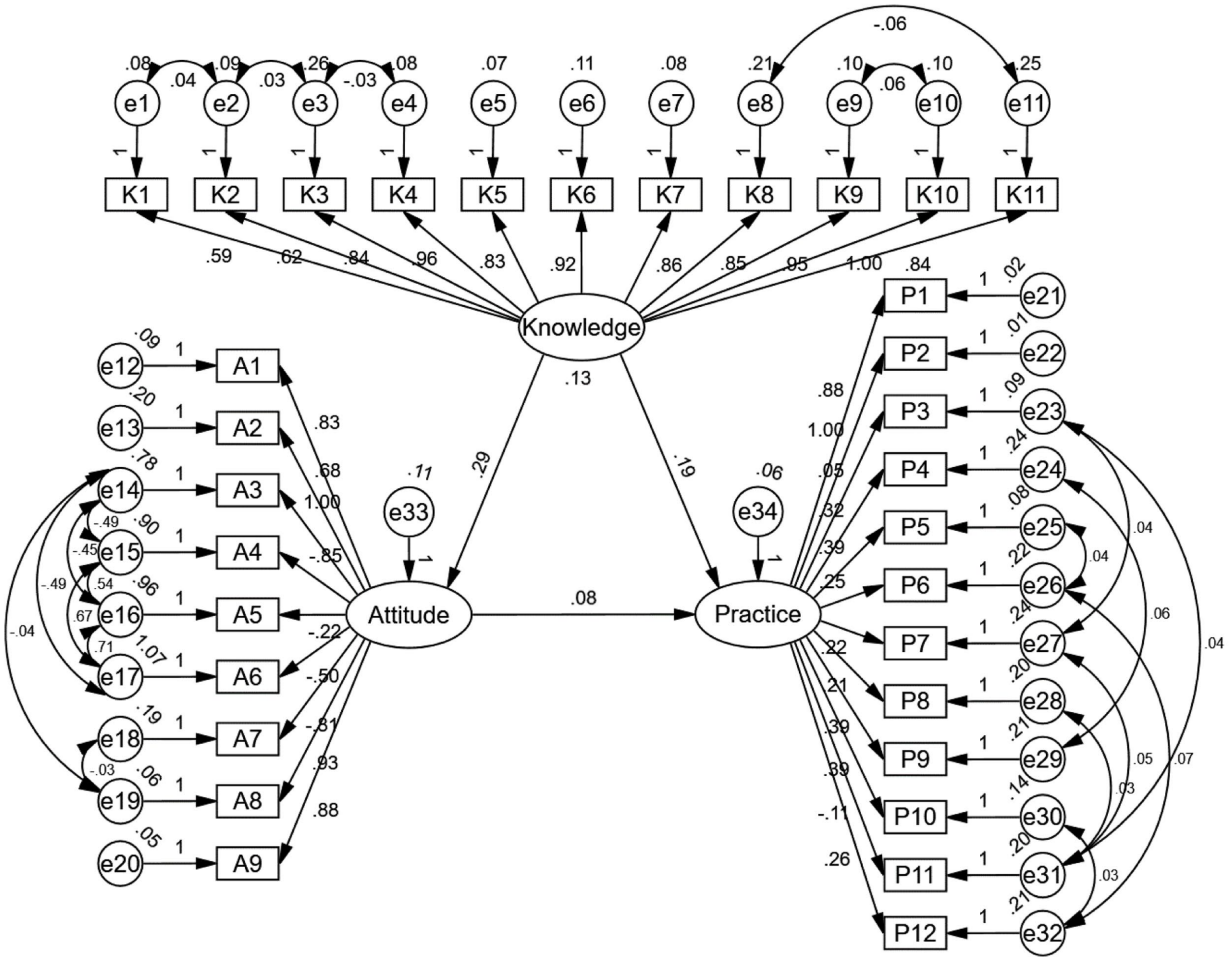
SEM model result.

The residual connection method was adopted to modify the SEM model so as to achieve a good fit (CMIN/DF value: 3.853, RMSEA value: 0.075, IFI value: 0.811, TLI value: 0.785, and CFI value: 0.810) (Supplementary Table S2), the results showed that knowledge had direct effects on attitude (*β* = 0.291, *p* < 0.001) and practice (*β* = 0.188, *p* < 0.001). Meanwhile, attitude had a direct impact on practice (*β* = 0.081, *p* = 0.045) (Supplementary Table S3 and Figure 1).

## Discussion

The study highlights a significant discrepancy among patients with RA, demonstrating inadequate knowledge but relatively positive attitudes toward dietary management, which did not strongly translate into active practice. It is recommended that clinical programs for RA patients incorporate targeted educational interventions to enhance dietary management knowledge, aiming to bridge the gap between attitudes and actual practices to improve health outcomes.

This study underscores a significant discrepancy between the positive attitudes toward dietary management in RA patients and their relatively insufficient knowledge and suboptimal practices. This finding is consistent with other studies indicating that while patients may be receptive to managing their condition through diet, a lack of comprehensive knowledge often hinders effective implementation (21, 22).

Specific factors such as average monthly income and education level significantly impacted KAP scores. For instance, participants with higher incomes exhibited better knowledge and practices, a pattern also reflected in the regression analysis where higher income was associated with improved knowledge. This finding suggests that economic factors may influence access to resources or educational opportunities that can enhance self-management capabilities (23, 24). Similarly, educational attainment correlated with knowledge and practice outcomes, reinforcing the need for tailored educational interventions that consider the varied socioeconomic backgrounds of patients.

Interestingly, while variables such as joint function classification and pain assessment significantly affected knowledge and attitudes, they did not markedly influence practice outcomes. This could be due to a lag between changes in perception and the actual adoption of new behaviors, or it may reflect other unmeasured barriers to behavior change such as physical limitations or psychosocial factors (25, 26).

The correlation and multivariate logistic regression analyses, as well as SEM, suggest a complex interplay between knowledge, attitudes, and practices. Our findings indicate that better knowledge about RA directly enhances both attitudes and practices, supporting the notion that informed patients are more likely to adopt and maintain beneficial dietary habits (27, 28). These results were consistent with previous research that emphasizes the pivotal role of patient education in chronic disease management (29, 30).

The findings from the knowledge dimension reveal substantial gaps in the understanding of dietary management among RA patients. A significant portion of the cohort displayed limited awareness of the role of BMI in RA risk, the importance of omega-3 fatty acids, and the specifics of anti-inflammatory diets such as the Mediterranean diet. This lack of knowledge was particularly pronounced concerning the impact of certain vegetables like potatoes, tomatoes, and eggplants on intestinal permeability and RA symptoms. To address these deficiencies, healthcare providers should implement targeted educational programs that not only explain the importance of diet in managing RA but also clarify common misconceptions. Programs could include detailed patient brochures, interactive online platforms, and structured dietary counseling sessions that tailor content to individual knowledge gaps and cultural preferences (31, 32).

Attitudinal responses highlighted a general recognition of the importance of diet management in RA, yet there was notable reluctance regarding its practical application and potential impact on quality of life. Particularly, many patients viewed diet management as cumbersome and less preferable compared to pharmacological treatments. To enhance acceptance and willingness to engage in dietary management, it is crucial to foster a supportive environment that empowers patients. This could be achieved through regular motivational interviewing sessions that address personal barriers and benefits perceived by patients, as well as through the development of peer-led support groups that provide empathy and practical tips from shared experiences. Furthermore, incorporating mobile health technologies that track dietary intake and provide real-time feedback could improve patients’ engagement and adherence (7, 33).

Practical adherence to recommended dietary guidelines was low, particularly in the usage of olive oil, the consumption of fish, fruits, and legumes, as well as in the reduction of red meat intake. This poor adherence might be reflective of both a lack of conviction in the efficacy of dietary measures and the perceived difficulty in integrating these into daily life (34, 35). To promote better dietary practices among RA patients, practical and accessible interventions are needed. This could include working with dietitians to create personalized meal plans that accommodate individual preferences and limitations, offering cooking classes that focus on preparing anti-inflammatory meals, and establishing partnerships with local food services to provide discounts on healthful food options. Additionally, implementing community-based programs that emphasize the social aspect of eating could help normalize and reinforce dietary changes (36, 37).

This study has several limitations that warrant consideration. First, the cross-sectional design limits the ability to establish causal relationships between the knowledge, attitudes, and practices regarding dietary management and rheumatoid arthritis outcomes. Second, the lack of a control group, as suggested, limits the ability to directly compare RA patients with a healthy population. Future studies should incorporate a quasi-experimental design to address this gap. In addition, the data were collected from a single institution, which may restrict the generalizability of the findings to broader populations with diverse healthcare settings and cultural backgrounds. Lastly, self-reported measures were used to assess dietary practices, which might introduce response biases and may not accurately reflect actual dietary behaviors.

## Conclusion

In conclusion, this study indicates that patients with RA demonstrate a discrepancy between a positive attitude toward dietary management and both insufficient knowledge and suboptimal practices. Significant predictors of better knowledge and attitudes include socioeconomic status, duration of RA, joint function, and type of medical insurance, which also influence dietary practices. These findings highlight the need for healthcare providers to integrate more robust dietary education into patient care. By offering clearer guidance on anti-inflammatory diets and providing continuous support, clinicians can help patients better manage RA symptoms, ultimately leading to improved treatment outcomes. Adjusting care plans to include routine dietary counseling may enhance the overall effectiveness of RA management.

## Data Availability

The original contributions presented in the study are included in the article/Supplementary material, further inquiries can be directed to the corresponding author.
