# Supplementary material for: Knowledge, attitudes, and practices of dietary management among patients with rheumatoid arthritis in China
Source: Front Public Health. 2024 Nov 8;12:1490189. doi: 10.3389/fpubh.2024.1490189 (PMC11582067; doi:10.3389/fpubh.2024.1490189)
Supplement: Supplementary file 1 [file Table_1.docx]

**Supplementary Table 1 (S1) Univariate and Multivariate Analysis Range**

|  | **Range** | **N (%)** |
| --- | --- | --- |
| **Knowledge score** | [0, 13.2] | 465 (92.26) |
|  | (13.2, 22] | 39 (7.74) |
| **Attitude score** | [9, 27] | 26 (5.16) |
|  | (27, 45] | 478 (94.84) |
| **Practice score** | [0, 7.2] | 460 (91.27) |
|  | (7.2, 12] | 44 (8.73) |

**Supplementary Table 2 (S2) SEM Model Fit**

| **Indicators** | **Reference** | **Actual** |
| --- | --- | --- |
| CMIN/DF | 1-3: Excellent, 3-5: Good | 3.853 |
| RMSEA | <0.08: Good | 0.075 |
| IFI | >0.8: Good | 0.811 |
| TLI | >0.8: Good | 0.785 |
| CFI | >0.8: Good | 0.810 |

**Supplementary Table 3 (S3) SEM Model Assumptions**

|  |  |  | **β** | **P** |
| --- | --- | --- | --- | --- |
| Attitude | <--- | Knowledge | 0.291 | <0.001 |
| Practice | <--- | Attitude | 0.081 | 0.045 |
| Practice | <--- | Knowledge | 0.188 | <0.001 |
| P1 | <--- | Practice | 0.877 | <0.001 |
| P2 | <--- | Practice | 1.000 |  |
| P3 | <--- | Practice | 0.051 | 0.352 |
| P4 | <--- | Practice | 0.318 | <0.001 |
| P5 | <--- | Practice | 0.394 | <0.001 |
| P6 | <--- | Practice | 0.249 | 0.004 |
| P7 | <--- | Practice | 0.225 | 0.013 |
| P8 | <--- | Practice | 0.213 | 0.010 |
| P9 | <--- | Practice | 0.393 | <0.001 |
| P10 | <--- | Practice | 0.391 | <0.001 |
| P11 | <--- | Practice | -0.107 | 0.188 |
| P12 | <--- | Practice | 0.259 | 0.002 |
| A9 | <--- | Attitude | 0.880 | <0.001 |
| A8 | <--- | Attitude | 0.934 | <0.001 |
| A7 | <--- | Attitude | -0.815 | <0.001 |
| A6 | <--- | Attitude | -0.501 | <0.001 |
| A5 | <--- | Attitude | -0.221 | 0.081 |
| A4 | <--- | Attitude | -0.848 | <0.001 |
| A3 | <--- | Attitude | 1.000 |  |
| A2 | <--- | Attitude | 0.679 | <0.001 |
| A1 | <--- | Attitude | 0.827 | <0.001 |
| K1 | <--- | Knowledge | 0.589 | <0.001 |
| K2 | <--- | Knowledge | 0.621 | <0.001 |
| K3 | <--- | Knowledge | 0.843 | <0.001 |
| K4 | <--- | Knowledge | 0.956 | <0.001 |
| K5 | <--- | Knowledge | 0.828 | <0.001 |
| K6 | <--- | Knowledge | 0.918 | <0.001 |
| K7 | <--- | Knowledge | 0.858 | <0.001 |
| K8 | <--- | Knowledge | 0.848 | <0.001 |
| K9 | <--- | Knowledge | 0.946 | <0.001 |
| K10 | <--- | Knowledge | 1.000 |  |
| K11 | <--- | Knowledge | 0.837 | <0.001 |
